# AR and YAP crosstalk: impacts on therapeutic strategies in prostate cancer

**DOI:** 10.3389/fonc.2025.1520808

**Published:** 2025-02-03

**Authors:** Guansong Zheng, Zhaojie Yan, Junrong Zou, Xiaofeng Zou, Keqiang Chai, Guoxi Zhang

**Affiliations:** ^1^ First Clinical College, Gannan Medical University, Ganzhou, China; ^2^ Department of Urology, First Affiliated Hospital of Gannan Medical University, Ganzhou, China; ^3^ Institute of Urology, Gannan Medical University, Ganzhou, China; ^4^ Department of Jiangxi Engineering Technology Research Center of Calculi Prevention, Gannan Medical University, Ganzhou, China; ^5^ Department of Urology, Third Affiliated Hospital of Gansu University of Chinese Medicine, Baiyin, China

**Keywords:** prostate cancer, androgen receptor, yes-associated protein, crosstalk, mechanism

## Abstract

Prostate cancer ranks as one of the most common types of cancer affecting men worldwide, and its progression is shaped by a diverse array of influencing factors. The AR signaling pathway plays a pivotal role in the pathogenesis of prostate cancer. While existing anti-androgen treatments show initial efficacy, they ultimately do not succeed in halting the advancement to CRPC. Recent studies have identified alterations in the Hippo-YAP signaling pathway within prostate cancer, highlighting intricate crosstalk with the AR signaling pathway. In this review, we examine the interactions and underlying mechanisms between AR and YAP, the key molecules in these two signaling pathways. AR regulates the stability and function of YAP by modulating its transcription, translation, and phosphorylation status, while YAP exerts both promotional and inhibitory regulatory effects on AR. Based on these findings, this paper investigates their significant roles in the onset, progression, and therapeutic resistance of prostate cancer, and discusses the clinical potential of YAP in prostate cancer treatment.

## 1 Introduction

Current statistical data indicates that prostate cancer is the most prevalent tumor found in the male urogenital system globally and ranks among the tumors with the highest number of survivors (1). The androgen receptor (AR) is an essential component in the prostate’s growth, development, and normal functioning (2, 3). It significantly contributes to the regulation of biological processes, including cell proliferation, growth, differentiation, and the cell cycle (4). Activated AR influences various cell signaling pathways by regulating gene transcription, thereby directly or indirectly affecting the function of prostate cells and male sexual differentiation (5). Furthermore, AR plays a pivotal role in the development of prostate cancer (6). On one hand, the abnormal activation or excessive expression of AR is closely linked to the pathophysiological alterations seen in prostate cancer. In the early stages, the AR signaling pathway maintains tissue homeostasis by controlling the proliferation and differentiation of normal prostate cells (7). However, when androgen levels increase or AR is abnormally amplified, the AR signaling pathway promotes the progression of prostate cancer (8). On the other hand, the progression of prostate cancer frequently involves changes in the AR signaling pathway, including AR mutations, gene rearrangements, and the reactivation of signaling pathways, which frequently leads to increased resistance of prostate cancer cells to androgen deprivation therapy (9, 10). Consequently, even with radical surgical resection combined with androgen deprivation therapy or anti-androgens, as well as radiotherapy, chemotherapy, and immunotherapy—current mainstream treatments (11) —this trend of tumors entering a resistant phase cannot be prevented, presenting a significant challenge in clinical treatment.

The Hippo signaling pathway was initially identified in Drosophila and is believed to modulate organ size and cellular differentiation by governing processes such as cell growth, programmed cell death, and the functionality of stem cells (12, 13). This pathway primarily consists of a serine-threonine kinase cascade module and the YAP/TAZ transcriptional module (14). Yes-associated protein (YAP) is a core component of the Hippo signaling pathway and also acts as a transcriptional co-activator (15). Overexpression of YAP is believed to be associated with various solid tumors (16–18). In prostate cancer, elevated levels of YAP expression are closely linked to tumor aggressiveness, growth capacity, and treatment resistance (19). A recent study summarized the roles and mechanisms by which transcription factors assist AR in promoting prostate cancer progression, proposing a therapeutic strategy aimed at targeting and silencing specific nodes within the transcriptional network (20).

In this review, we aim to summarize the interactions between AR and YAP in prostate cancer, while exploring their potential impacts on the disease’s development and treatment. We conducted a comprehensive analysis of the mechanisms underlying the interactions between AR and YAP, as well as the potential applications of YAP inhibitors in solid tumors. Through this research, we seek to enhance the understanding of YAP’s role in prostate cancer, providing new insights for related studies and offering more targeted decision-making support for researchers and clinicians in the design of treatment strategies.

## 2 Physical and functional interactions between AR and YAP

**Figure 1:**
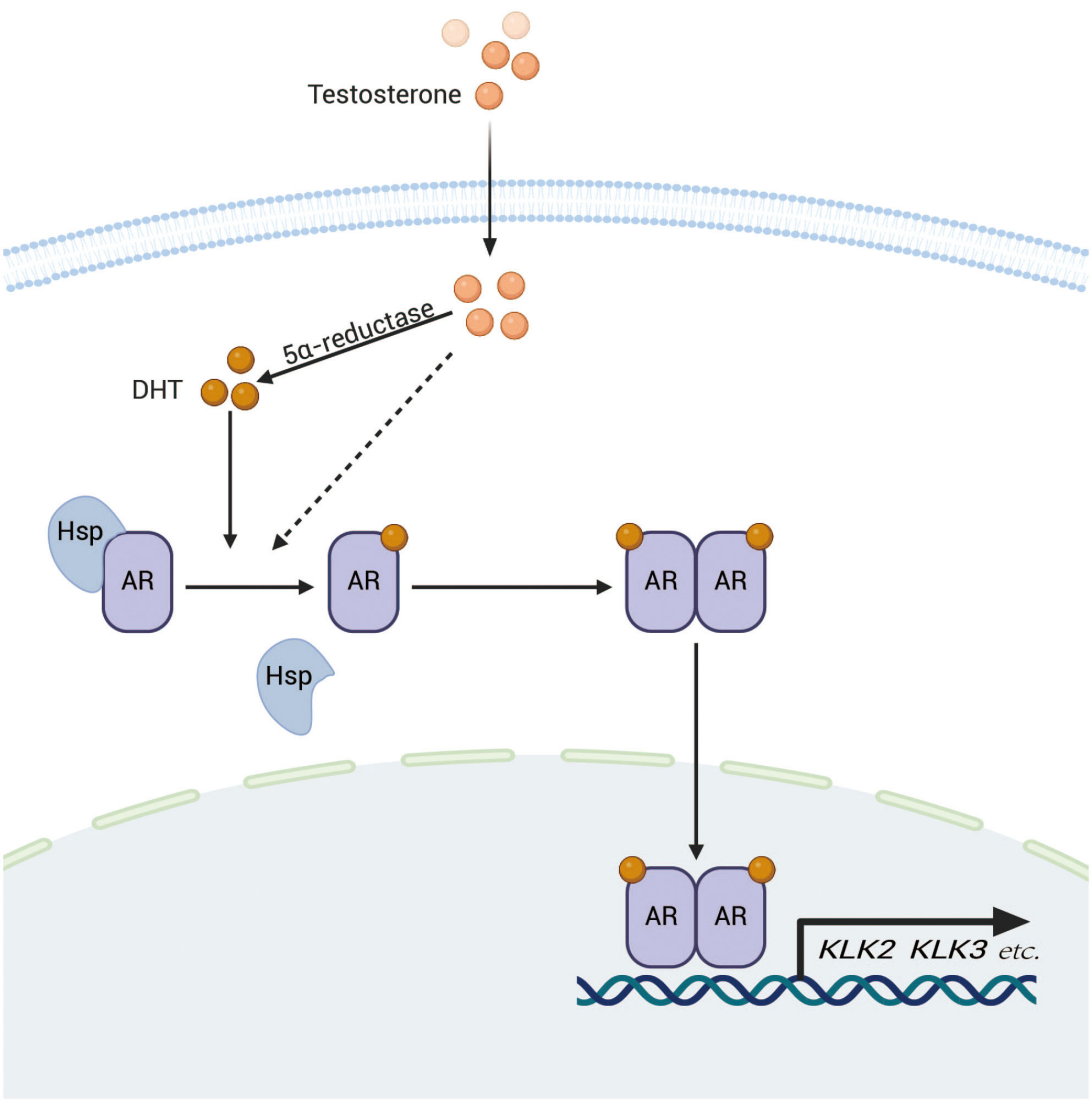
After entering the cell, testosterone is transformed into dihydrotestosterone (DHT) through the action of 5α-reductase.DHT subsequently binds to the AR, resulting in the release of AR from heat shock proteins and subsequent dimerization. The AR dimer then translocates to the nucleus, where it binds to ARE on DNA, thereby promoting the transcription of target genes such as KLK2 and KLK3, which are closely associated with the progression of prostate cancer.

The AR is part of the steroid receptor family and acts as both a nuclear receptor and a transcription factor (21). It has several functional regions, such as a transcriptional activation domain (TAD) located at the N-terminus, a domain for DNA binding (DBD), a hinge section, and a ligand-binding domain (LBD) at the C-terminus (22). In the cell, unactivated AR associates with heat shock protein 90 (hsp90) and is primarily localized in the cytoplasm. When androgens and other ligands enter the cell and bind to the, AR dissociates from the chaperone and becomes activated (23). Subsequently, the AR dimerizes and moves into the nucleus, where it interacts with androgen response elements (ARE) through the DNA binding domain (DBD), consequently modulating the transcription of target genes either by activation or repression (24) (**Figure 1**).

**Figure 2:**
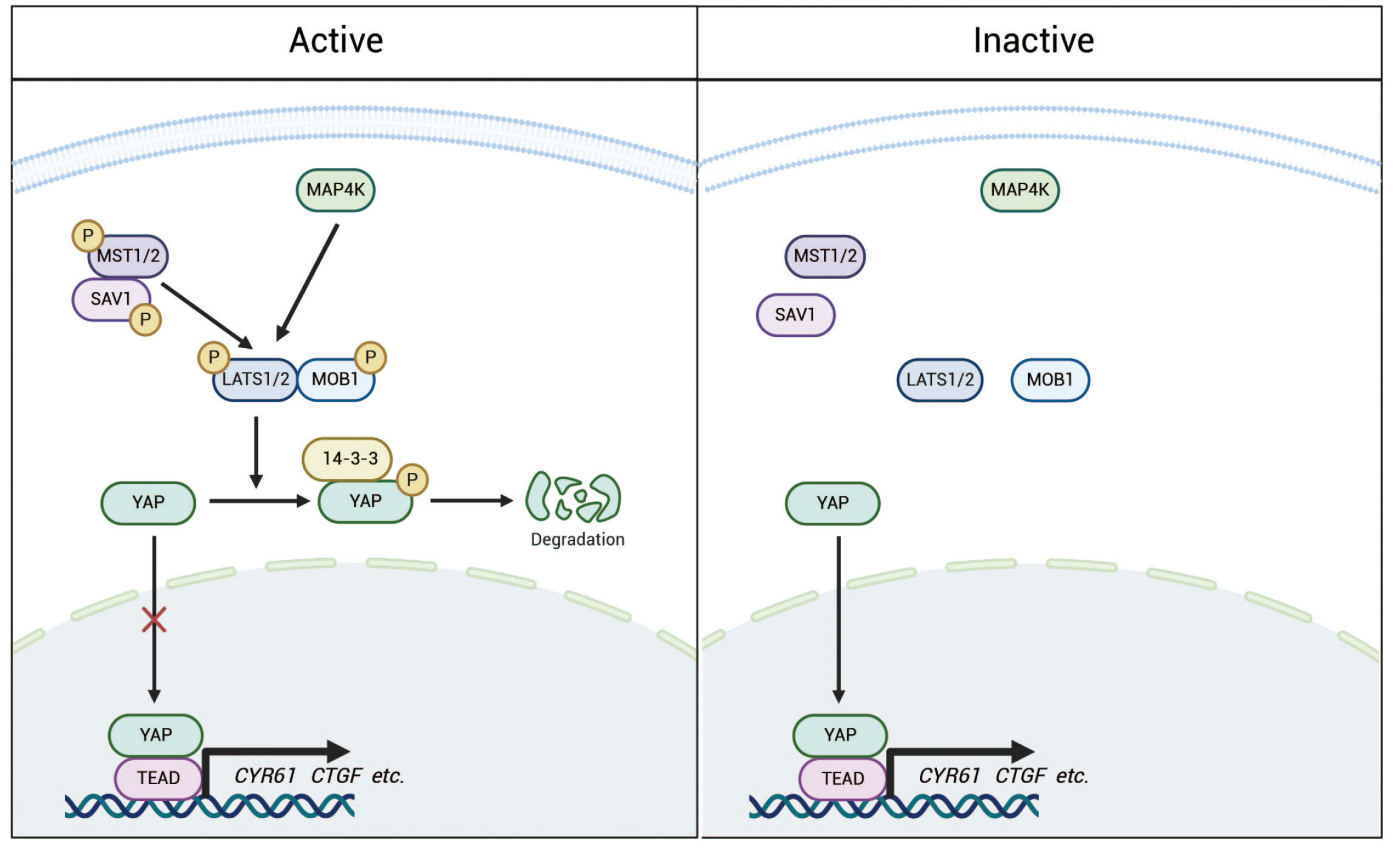
The Hippo pathway is a highly conserved signaling cascade composed of kinase modules and transcriptional regulators. Under normal conditions, the Hippo pathway remains active, with MAP4K and phosphorylated MST1/2 further phosphorylating LATS1/2. This phosphorylation subsequently leads to the phosphorylation of YAP, promoting its binding to 14-3-3 proteins and resulting in its retention in the cytoplasm for degradation. In contrast, when the pathway is inactivated, YAP becomes dephosphorylated and accumulates in the nucleus, where it interacts with TEADs and potentially other transcription factors to regulate the transcription of downstream target genes.

YAP is a core protein of the Hippo pathway and functions as a transcriptional co-activator (15). The Hippo pathway is a highly conserved signaling cascade composed of kinase modules and transcriptional modules (**Figure 2**), which together maintain cellular homeostasis (25). Upon activation of the pathway, MST1/2 (mammalian Ste20-like kinases 1/2) phosphorylate and activate LATS1/2 (large tumor suppressor kinases 1/2), which subsequently phosphorylate YAP, rendering it inactive. Inactive YAP loses its ability to enter the nucleus and instead binds to 14-3-3 proteins in the cytoplasm, leading to ubiquitin-mediated degradation (26). As a co-activator of transcription, YAP is not capable of directly binding to DNA. Consequently, upon entering the nucleus, activated YAP primarily regulates the expression of target genes, such as CYR61 and CTGF, by binding to transcriptional enhancer factor(TEAD) (27–29). Beyond its crucial role in maintaining adult tissue and organ homeostasis, the Hippo-YAP pathway also exerts significant effects on embryonic development in animals (30). Recent research has highlighted YAP’s critical role in stem cell self-renewal and differentiation (31), further supporting its therapeutic potential in organ regeneration and regenerative medicine (12).Abnormalities or inactivation of the Hippo pathway may lead to excessive activation of YAP. In prostate cancer, such excessive activation of YAP is associated with tumor progression, metastasis, and treatment resistance (26).

**Figure 3:**
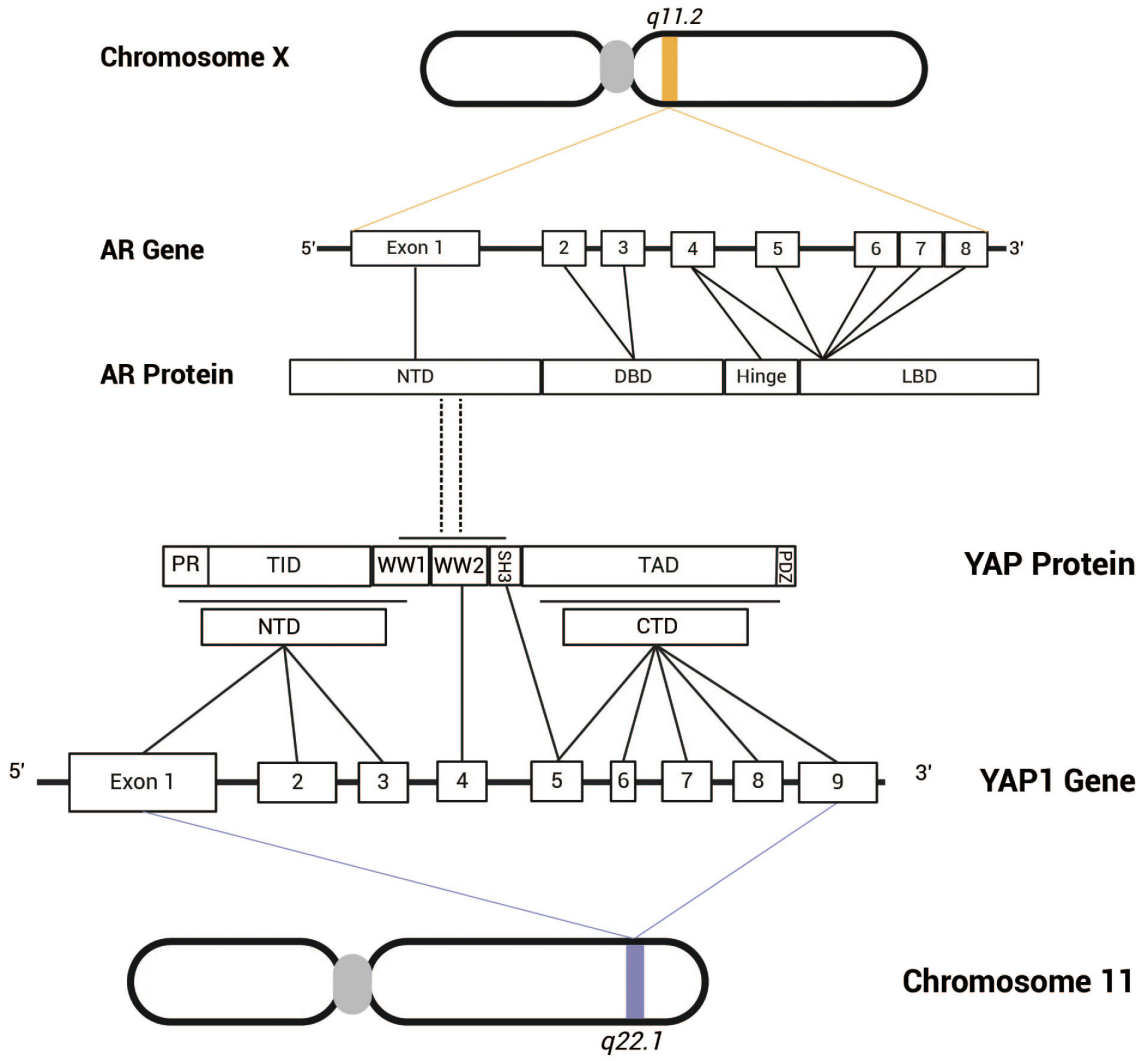
The androgen receptor gene is located on the X chromosome at q11.2 and comprises eight exons. The AR protein consists of several functional domains, including NTD, DBD, LBD. Exon 1 encodes the NTD, exons 2 and 3 encode the DBD, and exons 4 to 8 encode the hinge region and LBD. In contrast, the YAP gene is situated on chromosome 11 at q22.1 and contains nine exons. The YAP protein can be classified into two isoforms (YAP-1 and YAP-2) based on the number of WW domains. The NTD of YAP-2 features a proline-rich region (PR), a TEAD-binding domain (TID), and a WW-binding domain (WW1), which are encoded by exons 1-3, while another WW domain (WW2) is encoded by exon 4. Exons 5-9 encode the SH3-binding domain, transcriptional activation domain, and PDZ-binding motif. Research by Kuser-Abali et al. suggests that the WW/SH3 domain of YAP and the NTD of AR are likely the sites of interaction between the two proteins.

Through co-immunoprecipitation (Co-IP) and Western blot (WB) analyses, Kuser-Abali et al. demonstrated the presence of AR-YAP protein complexes in prostate cancer tissues, with heterogeneous expression levels across different samples. Furthermore, immunofluorescence and nuclear/cytoplasmic fractionation followed by Co-IP experiments confirmed that the AR-YAP interaction primarily occurs in the nucleus(32). The AR-YAP interaction exhibits distinct androgen dependency in different types of prostate cancer cells. Kuser-Abali et al. demonstrated through co-immunoprecipitation (Co-IP) and Western blot (WB) assays that this interaction is significantly enhanced in androgen-sensitive LNCaP cells upon stimulation with dihydrotestosterone (DHT) and markedly inhibited upon enzalutamide treatment, indicating androgen-dependent regulation. In contrast, in androgen-independent C4-2 cells (derived from LNCaP and acquired castration resistance), the AR-YAP interaction remains unaffected by either DHT stimulation or enzalutamide treatment, suggesting that the AR-YAP interaction has become independent of androgen presence (32). By dissecting the functional domains of YAP and AR and performing GST-pull-down experiments, the results indicate that the WW/SH3 domain of YAP and the NTD of AR are likely interaction sites for the two proteins (**Figure 3**). Kuser-Abali et al. also suggested that the differences in this interaction may be related to the expression level of MST1 in the cells. Specifically, MST1 can phosphorylate and activate LATS1/2, which subsequently phosphorylates YAP, preventing its translocation into the nucleus (33). Conversely, the study observed that MST1 can directly bind to YAP, promoting its phosphorylation and reducing YAP’s nuclear translocation, thereby directly decreasing the interaction between AR and YAP. Additionally, other studies suggest that MST acts as an inhibitor of androgen signaling by directly interacting with AR and diminishing its activity (34), which may also contribute to the reduced interaction between YAP and AR mediated by MST. Kuser-Abali et al. demonstrated that the suppression of YAP expression greatly reduced the proliferation and movement of prostate cancer cells as well as prostate cancer xenografts. This anti-proliferative effect was also noted in enzalutamide-resistant cell lines. Consequently, the interaction between AR and YAP constitutes a critical regulatory axis in cellular processes associated with prostate cancer, playing a vital role in the progression and metastasis of the disease (32). Moreover, complex regulatory relationships exist between AR and YAP at various molecular levels, which greatly affect the onset and progression of prostate cancer.

## 3 Diverse mechanisms underlying AR-mediated YAP regulation

### 3.1 AR affects YAP transcription through TMPRSS2-ERG

As prostate cancer progresses, the combination of PTEN and TP53 gene deletions and rearrangements of the ERG gene serves as a crucial driving factor (35–37) Among these alterations, the incidence of ERG rearrangement is the highest (38). The transcription factor known as ERG, which is produced by the ERG gene, was initially identified in colon cancer cells in 1987 and is recognized as a new member of the E-26 transformation-specific (ETS) oncogene family (39, 40). The ETS family of genes has been shown to play crucial roles as regulatory elements in the transcription process and are directly implicated in the mechanisms of cell proliferation, differentiation, and angiogenesis (41, 42). TMPRSS2 is a transmembrane protease found in normal prostate epithelium and is also present in semen (43). Both ERG and TMPRSS2 are located on chromosome 21, approximately 3 Mb apart (44, 45). Studies have found that in about 50% of prostate cancer cases, intronic deletions between the TMPRSS2 and ERG genes lead to the fusion of the TMPRSS2 promoter with ERG, a fusion that exists in over 90% of ERG-overexpressing prostate cancers (46). A recent observational study has also reported similar findings (47). TMPRSS2 is one of the target genes of the AR (48), and when androgens stimulate TMPRSS2-ERG fusion-positive prostate cancer cells, a significant increase in ERG expression can be observed (49). H3K9/14 acetylation is a chromatin mark that is highly localized to the 5’ regions of transcriptionally active human genes (50). Research by Nguyen et al. indicates that ERG can promote the expression of YAP in prostate cancer cells by influencing the H3K9/14 acetylation of the YAP promoter. Furthermore, the study revealed that silencing YAP effectively inhibited the trends of cell growth and tumor transformation induced by ERG overexpression in prostate epithelial cells. This indicates that YAP serves as an essential intermediate factor in the ERG-mediated transformation of prostate epithelial cells and the invasion of tumor cells (51). Recent studies have clearly established that the TMPRSS2-ERG fusion plays a significant role among the primary drivers of malignant transformation in normal prostate tissue (52). It is proposed that the TMPRSS2-ERG fusion event activates the proto-oncogenic properties of ERG within normal prostate epithelium (53). Moreover, the aberrant expression of ERG resulting from the TMPRSS2-ERG fusion is closely associated with increased cell proliferation, neovascularization, and invasive behavior in prostate cancer (54, 55). Based on the results of Nguyen et al., this phenomenon is likely mediated by YAP. In summary, AR modulates YAP expression through TMPRSS2-ERG, which has significant implications for the progression of prostate cancer.

### 3.2 The influence of AR on YAP translation

In addition to promoting the transcription of YAP, AR can also enhance its protein levels by influencing the translation process of YAP protein. Research conducted by Salem et al. observed that when androgens stimulated LNCaP cells, the protein level of YAP increased in correlation with the intensity and duration of stimulation, while no significant changes were noted in YAP mRNA levels (56). Furthermore, the study found that AR activation-induced increase in YAP activity was inhibited by the translation inhibitor cycloheximide (CHX) but was not affected by the proteasome inhibitor (MG132), indicating that AR activation influenced YAP’s translation process rather than protein degradation. Consequently, Salem et al. propose that AR promotes the aberrant activation of YAP by modulating the translation process of YAP protein, thereby influencing cell proliferation and metastasis. Although the precise mechanisms by which AR regulates protein translation in prostate cancer remain to be fully elucidated, existing studies suggest that AR may indirectly regulate the mTOR signaling pathway to participate in the protein translation process (57). Kallikrein-related peptidase 4 (KLK4) is one of the target genes of AR, which can enhance mTOR activity by inhibiting REDD1 (58). mTOR is a serine/threonine kinase that is categorized into two distinct complexes: mTOR complex 1 (mTORC1) and mTOR complex 2 (mTORC2) (59). Notably, mTORC1 can phosphorylate ribosomal protein S6 kinase and eukaryotic translation initiation factor 4E-binding protein (4EBP) (60). When eIF4E is phosphorylated and activated, it dissociates from 4EBP, enabling the formation of a heterotrimeric eIF4F complex in conjunction with scaffold protein eIF4G and RNA helicase eIF4A, thus facilitating mRNA recruitment to the ribosome (61, 62). The role of AR in protein translation may elucidate a potential mechanism through which AR contributes to prostate cancer progression by promoting YAP translation.

### 3.3 AR enhance stability and activity of YAP

AR not only upregulates protein levels by promoting the translation of YAP but also enhances the stability of the YAP protein. Studies have demonstrated that AR can enhance YAP protein stability and activity by modulating its post-translational modifications (63).

YAP, a core component of the Hippo pathway, is primarily localized in the cytoplasm, yet its primary transcription-dependent biological functions are typically executed in the nucleus. The translocation of YAP between the nucleus and the cytosol is regulated by multiple mechanisms, with post-translational modifications—especially phosphorylation—playing a crucial role in its nucleocytoplasmic transport (64, 65). YAP possesses multiple phosphorylation sites closely associated with its nuclear translocation, the most notable being the phosphorylation of serine-127. When the Hippo pathway is activated, MST1/2 and LATS1/2 kinases cascade to phosphorylate Ser-127, causing the phosphorylated YAP to bind with 14-3-3 proteins and sequester it in the cytoplasm, ultimately leading to its degradation via the ubiquitin-proteasome pathway (66, 67). Cinar et al. proposed the AR-STK4/MST1-PP2A axis as a regulatory mechanism for YAP nuclear localization (63), with serine/threonine phosphatases PP1/PP2A acting as effective inhibitors of STK4/3 (68). They demonstrated that androgen stimulation-induced AR activation in prostate cancer cells significantly reduced Ser-127 phosphorylation and increased both nuclear and total cellular YAP protein levels, thereby promoting the expression of YAP-dependent genes. These effects were abolished upon enzalutamide treatment or AR silencing (63). Additionally, ectopic expression of STK4/MST1 in cell lines confirmed that the reduction in Ser-127 phosphorylation levels resulted from antagonism between androgen signaling and STK4/MST1 (63). To determine whether androgen-induced inhibition of STK4/MST1 signaling was mediated by PP1/PP2A, Cinar et al. found that treatment with PP1/PP2A inhibitors restored Ser-127 phosphorylation levels (63). Therefore, it can be concluded that AR activation in prostate cancer cells may maintain YAP protein stability and activity by reducing Ser-127 phosphorylation levels.

## 4 Dual effects of YAP on AR signaling

### 4.1 The promoting effect of YAP on AR expression

In the progression of prostate cancer, evidence suggests that YAP can regulate AR, in addition to AR’s regulation of YAP. Bainbridge et al. demonstrated that inhibitor of nuclear factor kappa B kinase epsilon (IKBKE) could not only affect AR transcriptional levels through YAP but also regulate AR transcriptional activity, and proposed a regulatory strategy involving IKBKE-YAP-AR in prostate cancer (69). IKBKE, also known as IKK-I, is a mitogen-activated protein kinase and belongs to the non-classical IκB kinase family (70, 71). IKBKE plays a significant role in regulating inflammation, cellular immunity, and the progression of various metabolic diseases. It has been confirmed to be associated with multiple tumors, including prostate cancer (72–74). Bainbridge et al. found through WB and quantitative PCR experiments that silencing IKBKE not only inhibited the expression of AR target genes (such as PSA and TMPRSS2) in both androgen-sensitive and androgen-insensitive cells, but also significantly reduced the expression level of AR itself. Even with exogenous expression of AR-GFP, the expression of AR target genes was not restored. ChIP experiments further demonstrated that AR-GFP was still able to bind to the PSA enhancer, suggesting that IKBKE not only influences AR expression levels but also regulates its transcriptional activity (69). IKBKE has previously been shown to regulate YAP expression through its modulation of the Hippo pathway via LATS1/2 (75). Under IKBKE silencing conditions, Bainbridge et al. observed a significant reduction in the expression levels of both YAP and MYC. After treatment with the proteasome inhibitor MG132, the level of YAP was restored, further validating the role of IKBKE in regulating YAP expression (69). MYC, one of the most common oncogenes in humans, is a key transcription factor that plays an important role in various biological processes (76, 77). Dysregulation of MYC is often closely associated with the development and progression of multiple cancers (78, 79). Previous studies have indicated that MYC can directly bind to the AR gene, promoting AR mRNA synthesis (80). Bainbridge et al. observed a significant reduction in MYC binding to the AR gene in cells with silenced IKBKE, as shown by ChIP experiments. Moreover, ectopic expression of constitutively active YAP effectively restored the suppression of AR mRNA levels caused by IKBKE silencing (69). It is also noteworthy that MYC is considered a downstream target of YAP (81, 82), which further supports the conclusions of Bainbridge et al.’s study. Therefore, it can be concluded that in prostate cancer, YAP not only regulates AR expression but also does so in an IKBKE-dependent manner.

### 4.2 YAP’s suppression of AR signaling

While mainstream perspectives suggest that the synergy between YAP and AR promotes the development of prostate cancer, a recent study presents a contrasting viewpoint. This study argues that YAP competes with AR for TEAD, thereby inhibiting the expression of AR target genes (83). The TEAD, also referred to as transcription enhancer factors (TEFs), plays a critical role in development (84). Additionally, it is well-established that TEAD is associated with YAP (85). Using RNA-seq analysis, Li et al. discovered that in PCa cells with ectopic expression of YAP or constitutively active YAP, while YAP target genes were significantly upregulated, AR target genes were notably downregulated, and PCa cell proliferation was inhibited. Similar results were observed in cells treated with Hippo pathway inhibitors: AR target genes were downregulated in a dose-dependent manner, and this downregulation could be reversed by YAP knockdown (83). Further ChIP experiments indicated that this inhibitory effect might result from significantly reduced binding between AR and its target gene promoters/enhancers. Co-IP and WB experiments revealed that AR and YAP each formed complexes with TEAD1/4, although no direct binding between AR and YAP was detected. Meanwhile, ChIP-seq analysis demonstrated co-occupancy of AR and TEAD at AR target gene loci (83). Based on these findings, Li et al. proposed that YAP might suppress PCa progression by competing with AR for TEAD binding, thereby downregulating AR target genes. To test this hypothesis, researchers performed ChIP-qPCR analysis in cells expressing a YAP mutant deficient in TEAD binding. The results showed that neither AR binding to its target genes nor AR target gene expression levels were affected, further supporting their hypothesis (83). These findings suggest that YAP has the potential to suppress prostate cancer progression through inhibition of AR signaling.

Current research has noted that YAP can function as both an oncogene and a tumor suppressor in various cancers (86), although the specific regulatory mechanisms remain unclear. In prostate cancer and other tumors, the prevailing view still considers YAP as an oncogenic factor (87). While Li et al.’s findings differ from this mainstream perspective, they provide new insights into YAP’s dual regulatory role in AR signaling. Whether promoting or suppressing cancer development, YAP demonstrates significant value as a potential therapeutic target.

## 5 Clinical potential of YAP in tumor therapy

As the core protein of the Hippo signaling pathway, YAP is a promising and crucial potential drug target in a variety of solid tumors. Several drugs and compounds that inhibit YAP have already been developed or validated (88).

### 5.1 Verteporfin in preclinical glioblastoma models

Due to the absence of a DNA-binding domain, YAP cannot independently bind to DNA. Consequently, YAP must interact with DNA-binding transcription factors to associate with target gene promoters, thereby initiating the transcription of downstream genes and promoting cell proliferation, epithelial-mesenchymal transition (EMT), and the maintenance of stemness (89). Thus, the YAP-TEAD complex is considered central to the Hippo pathway and represents the most promising target for YAP inhibitors, particularly in comparison to the upstream proteins of the Hippo pathway. Verteporfin (VP), a benzoporphyrin derivative, was approved by the U.S. Food and Drug Administration (FDA) in 2000 for the treatment of macular degeneration (90). Recent studies have demonstrated that VP can selectively bind to YAP, inducing a conformational change that inhibits the interaction between YAP and TEAD (91). Glioblastoma (GBM) is recognized as the most aggressive and prevalent primary brain tumor, and it is largely regarded as an incurable disease. A recent analysis of chromatin accessibility in glioblastoma revealed that factors such as TEAD and YAP are associated with the migration and epithelial-mesenchymal transition of GBM (92). In the study conducted by Barrette et al., the effects of VP on tumor proliferation and migration *in vitro* were assessed using glioblastoma patient-derived cell lines, as well as its impact on tumor burden and survival rates in patient-derived xenograft (PDX) models (93).This study demonstrates that VP can disrupt the migration and epithelial-mesenchymal transition (EMT) of GBM by inhibiting YAP-TEAD activity. Furthermore, VP has been shown to reduce the burden of infiltrative tumors and improve survival rates in PDX models without causing systemic toxicity.

### 5.2 Super-TDU in the treatment of gastric cancer

Gastric cancer (GC) is the fifth most commonly diagnosed cancer and the fourth most common cause of cancer death (94). Studies have clearly indicated that the Hippo signaling pathway is abnormally expressed in various solid tumors, including GC, and have suggested that YAP is closely related to tumor growth and metastasis; thus, YAP is a critical therapeutic target in the treatment of GC (95). Vestigial-like family member 4 (VGLL4) is a transcriptional cofactor belonging to the VGLL family. Similar to YAP, VGLL4 lacks a DNA-binding domain and requires pairing with TEAD through its C-terminal Tondu (TDU) domain to exert its transcriptional regulatory functions (96–98). Research has demonstrated that VGLL4 is capable of directly competing with YAP for binding to TEAD, creating a complex with TEAD through its two TDU domains, which ultimately hinders the growth and advancement of cancer cells (99). Jiao et al. designed a VGLL4 mimetic peptide, Super-TDU, which can directly target TEADs and reduce the interaction between YAP and TEAD, resulting in a dose-dependent downregulation of the expression of YAP downstream genes CTGF, CYR61, and CDX2 (100). Furthermore, Jiao et al. demonstrated that Super-TDU can inhibit cell viability and colony formation of GC cell lines *in vitro*, as well as suppress the growth of GC tumors in mouse models. This further supports the potential of Super-TDU as a therapeutic agent for human cancers by inhibiting the YAP-TEAD interaction.

### 5.3 Hippo pathway activators in gastrointestinal tumors

The current development of YAP inhibitors primarily focuses on the relationship between Hippo-YAP dysregulation and tumor progression, which can be broadly categorized into two main strategies: 1. Activating the dysregulated Hippo pathway to inhibit YAP activation; 2. Directly targeting YAP/TEAD to prevent the formation of the YAP-TEAD complex (101). Although the strategy targeting the YAP-TEAD complex is more direct and effective, some studies have indicated that Hippo pathway-activating drugs show promise in gastrointestinal tumors. Dysregulation of the Hippo pathway is prevalent in tumors (102). Among the various mechanisms, the inactivation of MST1/2 and LATS1/2 is often implicated in the abnormal activation of YAP, leading to excessive proliferation and tumorigenesis (103). Tang et al. discovered that STRN3 functions as a modulator of PP2A, facilitating the recruitment of MST1/2 to the PP2A core enzyme complex, which results in the dephosphorylation of MST1/2 (104). Building on this finding, Tang et al. developed a Hippo-activating peptide (SHAP) derived from STRN3. SHAP disrupted the interaction between STRN3-mediated MST1/2 and PP2A in a dose-dependent manner. In PDX models of gastric cancer, significant tumor regression was observed, accompanied by a notable reduction in the expression of YAP target genes (CYR61, CTGF). Furthermore, RNA-seq transcriptome analysis and GSEA demonstrated that Hippo signaling is a target of SHAP. A recent study suggested that other drugs with similar mechanisms of action can reactivate the Hippo pathway, thereby inhibiting gastric cancer and enhancing chemotherapy sensitivity (105).MST1/2 activation drugs not only effectively inhibit the progression of gastric cancer but are also capable of significantly suppressing the metastasis of colorectal cancer. Tripartite motif-containing protein 21 (TRIM21) is a widely distributed RING-type E3 ubiquitin ligase (106). In colorectal cancer, TRIM21 can directly interact with and ubiquitinate the lysine 473 (K473) site of MST2 via K63 linkages (107). This ubiquitination enhances the formation of MST2 homodimers, which are critical for its autophosphorylation and activation (108, 109). Activated MST2 inhibits the epithelial-mesenchymal transition (EMT) of tumors by influencing the nuclear-cytoplasmic translocation of the YAP protein. Liu et al. identified Vilazodone as an ideal ligand for TRIM21 using the AlphaFold and ZINC15-DrugBank databases, demonstrating its effectiveness in inhibiting the migration and invasion of colorectal cancer (CRC) cells. In summary, the therapeutic strategy of reactivating the Hippo pathway has shown promise in the treatment of gastrointestinal tumors, providing further insights into the clinical potential of YAP inhibitors.

### 5.4 YAP as a target in prostate cancer treatment

YAP serves as a critical factor in prostate cancer progression. Studies have shown that silencing YAP significantly inhibits tumor cell proliferation, metastasis, and castration resistance (26, 110, 111). However, there are currently no clinical studies on YAP inhibitors related to prostate cancer. Clemens Thoma suggested that targeting the AR-YAP interaction might be key to treating this disease (112). Kuser-Abali et al. silenced YAP expression in C4-2 cells using shRNA technology and found that compared to the control group, the growth and invasion abilities of the shYAP group were significantly reduced. Moreover, both androgen-induced cell proliferation and AR target gene expression were suppressed, and this result was confirmed in a mouse model (32). In VP-treated cells, besides inhibiting cell proliferation and invasion, apoptosis was also promoted. Immunofluorescence, Co-IP, and WB experiments confirmed that VP significantly inhibited YAP nuclear translocation and the AR-YAP interaction (32).YAP not only plays a crucial role in tumor cell proliferation and invasion but has also been demonstrated to induce cancer stemness in prostate cancer, thus promoting enzalutamide resistance (113). In a mouse model constructed with enzalutamide-resistant cells by Hsiu-Chi Lee et al., VP showed significantly better tumor growth inhibition than other AR inhibitors (113). In the tumor microenvironment, studies have found that YAP can promote the recruitment of myeloid-derived suppressor cells (MDSCs), thereby exerting immunosuppressive effects. Silencing YAP expression in a mouse model reduced MDSC infiltration and inhibited tumor growth (114). Recent studies have also proposed a therapeutic approach combining YAP inhibition, radiotherapy, and PD-1 blockade. Although this strategy was tested in a preclinical colon cancer model, it provides new evidence for the immunomodulatory effects of YAP inhibitors and presents new insights for combination therapy approaches (115).

In conclusion, the multiple roles of YAP in prostate cancer make it a potential therapeutic target, and future clinical studies are expected to reveal its practical efficacy as a treatment strategy.

## 6 Conclusions and prospects

With the in-depth research on prostate cancer, the unequivocal central role of AR in this disease has been underscored, while the significant role of the Hippo-YAP pathway has also gained considerable attention. Existing studies have clearly indicated that YAP is closely associated with the occurrence, progression, and invasiveness of prostate cancer; however, its complex mechanisms remain poorly understood. This review offers a novel perspective on prostate cancer research by exploring the interactions and related mechanisms between AR and YAP in this context. The research includes the direct binding of AR to YAP in the nucleus, the abnormal transcription of YAP induced by AR through TMPRSS2-ERG, and the influence of AR on YAP protein stability through processes such as protein translation and post-translational modifications. In addition to the regulation of YAP by AR, this review also examines the dual regulatory effects of YAP on AR transcriptional activity, encompassing both promotion and inhibition. By integrating existing research findings, I posit that the regulation of YAP by AR constitutes a primary regulatory mechanism in prostate cancer, whereas the regulation of AR by YAP operates more as a coordinating feedback control, with both factors influencing key biological processes such as cell proliferation and migration.

While this review provides valuable insights into the mechanisms of YAP and AR in prostate cancer, several critical issues within the current research on prostate cancer remain unaddressed. First, the molecular mechanisms underlying YAP-AR interactions remain to be fully elucidated. Furthermore, there is still no consensus regarding YAP’s dual regulatory effects on AR signaling, which is crucial for developing YAP-targeted therapeutic strategies in prostate cancer. Future studies are anticipated to validate these interactions across various types of prostate cancer samples and datasets, thereby providing clearer insights into prostate cancer research. Furthermore, this review highlights the clinical potential of YAP in tumor therapy, discussing the promising efficacy of YAP direct inhibitors and Hippo pathway activators in tumors. The limitations of current research are also noted, for instance, while certain YAP inhibitors demonstrate promise in preclinical models of specific tumors, there is a notable lack of clinical evidence regarding their efficacy and safety in cancer treatment. Additionally, despite YAP’s significant role in prostate cancer, there is a dearth of therapeutic research targeting YAP. Currently, the standard treatment for prostate cancer involves surgery combined with anti-androgen therapy; however, this approach often leads to resistance and late-stage metastasis. Given the discussions in this review regarding the crosstalk mechanisms between AR and YAP in prostate cancer, we propose that future research could investigate new targeted drugs against YAP and develop more specific therapeutic strategies. For example, combining YAP inhibitors with androgen deprivation therapy may effectively suppress AR signaling, inhibit tumor progression, delay the onset of castration resistance, and enhance patient survival and quality of life. For patients who have developed castration resistance, the incorporation of YAP inhibitors may help to restore tumor sensitivity to related anti-androgen therapies.
